# Methodology and results of integrated WNV surveillance programmes in Serbia

**DOI:** 10.1371/journal.pone.0195439

**Published:** 2018-04-06

**Authors:** Tamaš Petrović, Milanko Šekler, Dušan Petrić, Sava Lazić, Zoran Debeljak, Dejan Vidanović, Aleksandra Ignjatović Ćupina, Gospava Lazić, Diana Lupulović, Mišo Kolarević, Budimir Plavšić

**Affiliations:** 1 Department for virology, Scientific Veterinary Institute “Novi Sad”, Novi Sad, Serbia; 2 Specialized Veterinary Institute “Kraljevo”, Kraljevo, Serbia; 3 Laboratory for medical and veterinary entomology, Faculty of Agriculture, University of Novi Sad, Novi Sad, Serbia; 4 Ministry of Agriculture and Environmental protection, Veterinary Directorate, Belgrade, Serbia; Keele University Faculty of Natural Sciences, UNITED KINGDOM

## Abstract

Studies conducted during the past few years have confirmed active West Nile virus (WNV) circulation in Serbia. Based on these studies and the epidemiological situation, the Veterinary Directorate of the Ministry of Agriculture and Environmental Protection launched national WNV surveillance programmes in 2014 and 2015. The programmes encompassed the territory of Serbia and were conducted by the veterinary service in collaboration with entomologists and ornithologists. The objective of the programmes was early detection of WNV and timely reporting to the public health service and local authorities to increase both clinical and mosquito control preparedness. The WNV surveillance programmes were based on direct and indirect surveillance of the presence of WNV by the serological testing of initially seronegative sentinel horses and chickens as well as through viral detection in pooled mosquito and wild bird samples. The most intense WNV circulation was observed in all seven districts of Vojvodina Province (northern Serbia) and Belgrade City, where most of the positive samples were detected among sentinel animals, mosquitoes and wild birds. The West Nile virus surveillance programmes in 2014 and 2015 showed satisfactory results in their capacity to indicate the spatial distribution of the risk for humans and their sensitivity to early detect viral circulation at the enzootic level. Most of the human cases were preceded by the detection of WNV circulation as part of the surveillance programmes. According to the existing data, it can be reasonably assumed that WNV infection, now an endemic infection in Serbia, will continue to present a significant problem for the veterinary service and public health.

## Introduction

West Nile virus (WNV) is a zoonotic neurovirulent mosquito-borne *Flavivirus*, which is maintained in nature in an enzootic transmission cycle between avian hosts and ornithophilic mosquito vectors. The virus occasionally infects other vertebrates, including humans and horses that are incidental, dead-end hosts and in which it may cause sporadic disease outbreaks that may result in fatal outcomes. Today, WNV is considered one of the most widespread flaviviruses in the world and is endemic in Africa, Asia, Europe, the Middle East, Australia and the Americas [1,2].

In Europe, until the 1990s, WNV caused sporadic outbreaks with rare reports of encephalitis, but its epidemiological behaviour changed when it re-emerged in Romania, Russia and the Mediterranean basin, causing dozens of human and equine deaths [1,3]. Additionally, only recently have strains belonging to WNV lineage 2 been identified in Europe: in 2004 and 2005 in goshawks and birds of prey in Hungary, in 2007 in Volgograd, Russia, and in 2008 and 2009 in goshawks and a falcon in Austria [4–7]. Since 2008, WNV has been heavily spreading throughout central and south-eastern Europe and constituting a serious veterinary and public health problem for Europe [8,9].

In Serbia, the WNV situation was mostly unknown until 2009. Serological testing of horses sampled during 2009–2010 showed for the first time in Serbia that 12% of 349 horses from the northern part of the country had neutralizing WNV antibodies. WNV antibody positive horses were found in 14 out of 28 municipalities studied, spread over 200 km [10]. In another study, the presence of specific antibodies against WNV was found in 28.6% (72) out of 252 examined horse sera samples collected from 7 large horse stables located in Vojvodina Province (northern part of Serbia–NUTS2 level) and the City of Belgrade District (NUTS3 level) during 2007–2011 (The NUTS classification is a hierarchical geocode standard in EU for dividing up the subdivisions of countries for statistical purpose. There are three levels of NUTS that refer to the major socio-economic regions—NUTS1 (country), NUTS2 (province) and NUTS3 (district)). Seroprevalence ranged per stable from 13.3% up to 40% [11]. In addition, just one year later, to assess WNV presence in the environment immediately after the human WNV outbreak in 2012, the presence of WNV IgG antibodies was detected in 64 (49.23%) out of 130 tested blood sera samples of horses from 6 stables and 1 settlement in Vojvodina Province. Per stable, the percentage of seropositive animals varied from 35% to 64% [12].

The first studies of the presence of WNV in mosquito vectors in Serbia date back to the period 2005–2010. A total of 56,757 mosquitoes (841 pools of 50 individual insects) originating from 66 localities in 29 settlements in Vojvodina Province were examined. West Nile virus RNA was detected in only 3 out of 841 mosquito pools tested. West Nile virus positive pools were found in the city of Novi Sad in 2010, and the sequenced isolate was typed as WNV lineage 2 [13]. These studies were also continued during 2012 and 2013, when a significant increase in the prevalence of WNV in mosquitoes was established. More than 9% of the mosquito pools examined during 2012–2013, mainly of the species *Culex pipiens* (both biotypes *molestus* and *pipiens* and their hybrids), tested positive for WNV presence (unpublished data).

In addition, the first study of WNV presence in wild birds as natural viral hosts confirmed intensive WNV circulation in Serbia during 2012. WNV antibodies were detected in 7.6% (7/92) of the blood sera samples, and the presence of the virus was confirmed in 8 out of 81 (9.87%) tissue samples and in one bird blood sample out of 133 dead and live captured wild resident and migratory birds sampled and tested from January until September 2012 from Vojvodina Province. Most of the birds positive for WNV antibodies or viruses were strictly residential, suggesting the endemic presence of WNV in Serbia. All WNV isolates were typed as lineage 2 strains [14].

The history of WNV infection among the human population in Serbia is mostly unknown due to the lack of routine diagnostic testing, prior to 2012, for human cases of meningoencephalitis with unknown origin. The first serological studies conducted among human populations in Serbia showed a low prevalence for WNV antibodies, ≤8% from the early 1970s to 1990 [15,16], indicating possibly low or sporadic circulation of WNV in the past in Serbia. After a gap of many years, more recent serological examinations revealed the presence of WNV IgG antibodies in 18 out of 451 (3.99%) human sera samples collected from 2005 to 2010 in Vojvodina, with yearly rates varying between 1.97% and 6.04% [13]. In 2012, an outbreak of WNV infection in humans was reported for the first time in Serbia [17,18]. It was the first time that WNV infections in the country were associated with recorded clinical symptoms. A total of 69 West Nile fever cases were reported, among which 42 were clinically and laboratory confirmed, and 9 cases were fatal. All cases were detected in the central and northern parts of the country [18,19]. This epidemic continued, and it became even more severe during 2013. A total of 302 West Nile fever cases were reported in 2013, among which 202 were laboratory confirmed and 35 cases were fatal (lethality of 11.6%). Almost all cases were again detected in the central and northern parts of the country [20].

All aforementioned research studies confirmed the active circulation and endemic presence of WNV in the territory of Serbia. With the exception of monitoring conducted for research purposes, regular programme-based WNV surveillance did not exist before 2014. Based on the obtained results and the anticipated intense circulation of WNV, which poses substantial risks for both public and animal health in Serbia, the Veterinary Directorate of the Ministry of Agriculture and Environmental Protection launched and funded national WNV surveillance programmes in 2014 and 2015. The methodology of the implementation and management of these surveillance programmes as well as the data obtained are presented in this article.

## Material and methods

### Methodology of WNV surveillance programs in Serbia

WNV national surveillance programmes, established by the Veterinary Directorate of the Serbian Ministry of Agriculture and Environmental Protection as both active and passive surveillance, have been conducted over the course of two consequent seasons (2014 and 2015). In the preparation of the WNV surveillance programmes (first in 2014 and second in 2015), existing knowledge about WNV transmission and epidemiology was incorporated, and the parameters of different programme models [9,21–24] were studied. In addition, existing guidance for the surveillance, prevention and control of WNV infection was used [25]. The main objective of the programmes were the early detection of WNV circulation in nature and the consequent provision of timely information to veterinary services and human epidemiologists for the purpose of establishing protective measures for human and animal health and guiding mosquito control efforts. The surveillance programmes were based on direct and indirect monitoring of the presence of WNV in nature. Horses and backyard chickens were used as sentinel animals for serology testing (as indirect indicators of viral presence), whereas wild birds and mosquitoes were tested for the presence of the virus by molecular methods as part of an active surveillance programme. Passive surveillance encompassed serological (testing of paired serum samples) and virological examinations (RT-qPCR) of clinically ill horses manifesting signs of neurological dysfunction.

The programmes encompassed the entire territory of Serbia and were conducted by veterinary institutes and field veterinary services in close collaboration with entomologists and ornithologists. The selection and distribution of sampling localities for active surveillance in each district were defined according to the assessment of the risk of exposure to WNV. In assessing the exposure risk, the following was taken into consideration:

1. already available serological results from horses;
2. the existence of areas suitable for mosquito breeding;
3. settlements with previous histories of human infections.

**Fig 1:**
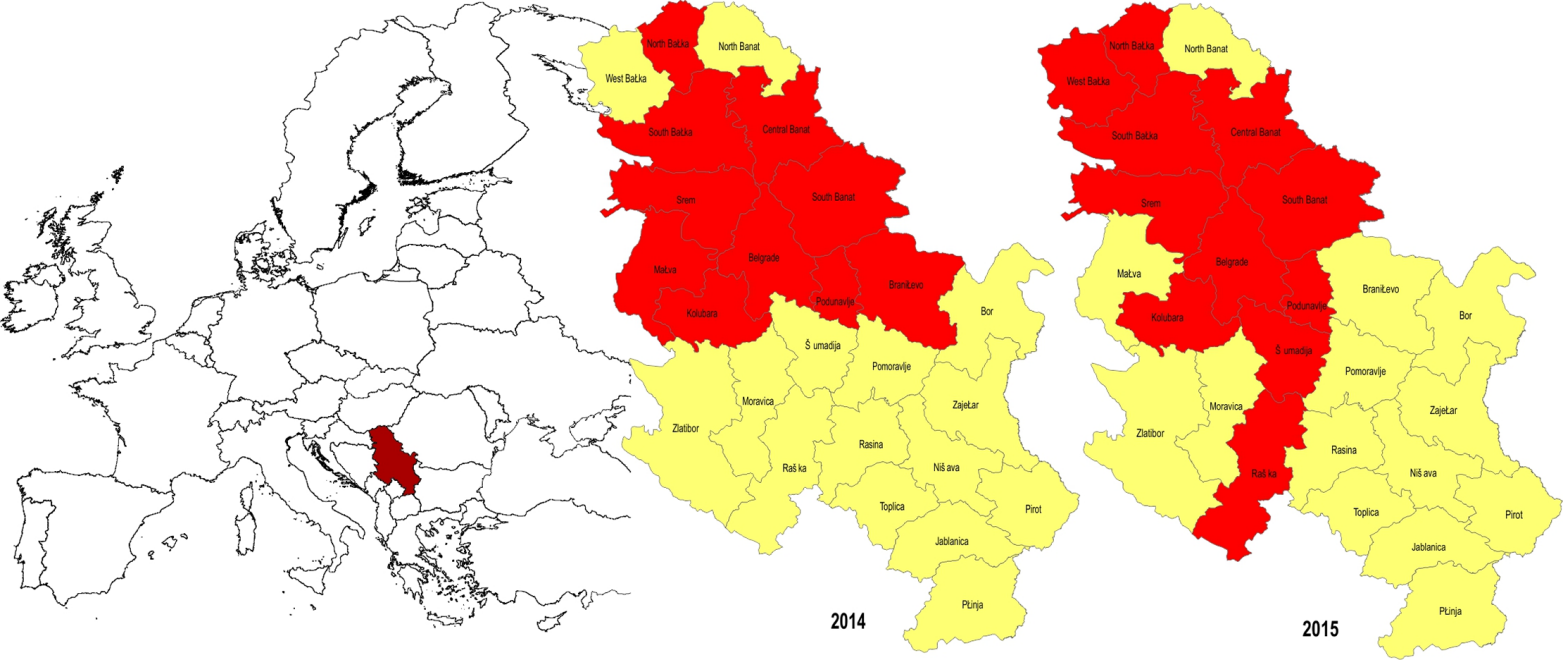
Categorization of districts in the Republic of Serbia according to risk of WNV outbreak. The figure show the geographic position of Republic of Serbia in Europe. The districts (NUTS3) with higher risk of WNV infection are marked in red (10 districts in WNV surveillance programme in 2014 and 11 districts in WNV surveillance programme in 2015), and the districts with lower risk of WNV infection are marked in yellow. The solid black lines in the maps of Republic of Serbia represents the borders of districts (25 districts in Serbia that were under WNV surveillance in 2014 and 2015). The northernmost seven districts represent the Autonomous Province of Vojvodina. Description of the sampling locations: Districts names, geographic coordinates and web links are provided as the supporting information S3 Table. The basic administrative maps were extracted from the GADM database of Global Administrative Areas (www.gadm.org), version 2.8, November 2015, and later on changed according to the data presented in them.

According to the previous results regarding WNV presence in horses, wild birds and mosquitoes as well as human outbreaks during 2012, 2013 and 2014, the territory of Serbia was divided into districts with higher or lower risk of WNV presence in surveillance programmes conducted during 2014 and 2015 (25 districts–NUTS3 level, Fig 1). The number of tested samples was defined at the level of each district in relation to the risk of WNV infection.

### Serological surveillance of sentinel chickens

Serological surveillance of backyard chickens was only performed in 2014. Only backyard chickens that were hatched in 2014 (during the year of testing and not earlier to exclude animals that were seropositive from previous years) were used for WNV surveillance. Sampling was mainly conducted in suburban areas as outlined in Table 1. Throughout the entire surveillance period (from June until September), blood samples were collected at the same locations for all sampling occasions. The term “location” refers to the area of selected settlements. Immediately upon sampling, the collected samples were tested for the presence of WNV IgG antibodies by a commercial blocking ELISA kit (*INGEZIM WEST NILE COMPAC*, Ingenasa, Spain) following the manufacturer’s instructions.

**Table 1:** WNV surveillance plan (sampling and testing) in 2014.

	Lower-risk districts												High-risk districts											
**Testing of sentinel animals (backyard chickens and horses) for WNV IgG antibodies (ELISA)**																								
Sentinel chickens*	- from 6 settlements per district; - 5 samples per settlement - 4 samplings (1 in June; 1 in July; 1 in August and 1 in September)												- from 10 settlements per district - 5 samples per settlement - 6 samplings (2 in June; 2 in July; 1 in August and 1 in September)											
Sentinel horses**	- up to 30 sentinel horses - minimum 3 localities per district - testing of the same horses in three occasions (June-July-August)												- up to 50 sentinel horses - minimum 3 localities per district - testing of the same horses in three occasions (June-July-August)											
**Real-time RT-PCR detection of WNV in natural reservoirs and vectors**																								
Wild birds	- up to 50 found dead wild birds from May—October												- all found dead throughout the year, or - up to 100 samples of purposely shot or live trapped susceptible bird species from May to October											
Mosquitoes (*Culex pipiens*)	- collection at monthly intervals - 5 localities per district - 5 samplings from May to September												- collection at 2-week intervals - 10 localities per district - 7 samplings from May to September											
**Sampling strategy**																								
Sampling distribution	Jan	Feb	Mar	Apr	May	Jun	Jul	Aug	Sep	Oct	Nov	Dec	Jan	Feb	Mar	Apr	May	Jun	Jul	Aug	Sep	Oct	Nov	Dec
Chickens																								
Horses																								
Mosquitoes																								
Wild birds																								

* Surveillance of rural, backyard chickens was limited to chickens hatched in 2014

** Sentinel horses were limited to horses previously tested negative for WNV IgG antibodies

### Serological surveillance of sentinel horses

During spring 2014 (April-May), blood sera samples from horses in each district were tested for the presence of WNV IgG antibodies by a commercial blocking ELISA kit (*INGEZIM WEST NILE COMPAC*, Ingenasa, Spain) following the manufacturer’s instructions. Only the animals that tested negative for WNV IgG antibodies were used as sentinel animals for WNV surveillance during the 2014 season. Sentinel horses were sampled as described in Table 1 and tested for the presence of WNV IgG antibodies using the aforementioned ELISA kit. Thus, the same sentinel animals were sampled and tested three times during the three surveillance months in 2014. In contrast, during the 2015 season, a large number of already seropositive horses caused difficulties in finding an adequate number of seronegative sentinel horses to be included in the surveillance, so the serology surveillance in horses was performed by detection of WNV IgM antibodies (instead of IgG as in 2014). The horses were sampled as described in Table 2 and tested for the presence of WNV IgM antibodies by a commercial ELISA test (*ID Screen West Nile IgM Capture*).

**Table 2:** WNV surveillance plan (sampling and testing) in 2015.

	Lower-risk districts												High-risk districts											
**Testing of sentinel horses for WNV IgM antibodies (ELISA)**																								
Sentinel horses	- up to 30 horses - minimum of 7 localities per district - testing on four occasions (one per month in June, July, August and September)												- up to 50 horses - minimum of 10 localities per district - testing on four occasions (one per month in June, July, August and September)											
**Real-time RT-PCR detection of WNV in natural reservoirs and vectors**																								
Wild birds	- up to 50 dead wild birds found during the period of May—October												- all found dead throughout the year, or - up to 100 samples of purposely shot or live-trapped susceptible bird species during the period of May—October											
Mosquitoes (*Culex pipiens*)	- collection at monthly intervals - 5 localities per district - 5 samplings from June to September (2 samplings in July)												- collection at 2-week intervals - 10 localities per district - 7 samplings from June to September											
**Sampling strategy**																								
Sampling distribution	Jan	Feb	Mar	Apr	May	Jun	Jul	Aug	Sep	Oct	Nov	Dec	Jan	Feb	Mar	Apr	May	Jun	Jul	Aug	Sep	Oct	Nov	Dec
Horses																								
Mosquitoes																								
Wild birds																								

### WNV surveillance in wild birds

Dead wild birds found in the natural environment, particularly the resident species most susceptible to infection, e.g., from the order Passeriformes, mainly *Corvidae* (magpie, crow, raven, rook., etc.), raptors (northern goshawk, falcon and eagle), common pheasants and song birds, as well as birds that died in rehabilitation centres, zoos or bird breeding farms (mostly raptors such as falcons, eagles, and hawks), were submitted for testing of the presence of WNV. Mentioned wild bird species were previously confirmed as bird species in which WNV was very often detected in previous years in this part of Europe [4,14,26]. Tissues from wild birds found dead and wild birds that were shot (corvids—mainly Eurasian magpies) during surveillance and tracheal / pharyngeal swabs of live-trapped wild birds were tested for the presence of WNV as described in Tables 1 and 2.

### WNV surveillance in mosquitoes

Mosquitoes were sampled from May to September, by dry-ice baited traps without light (trap types NS2 and EVS), operating overnight (in average 14 hours, from afternoon to the next day morning), in semi-urban and urban localities, as described in Tables 1 and 2. Mosquitoes were collected, put on dry ice in the field and maintained under cold conditions throughout the testing process. They were identified to the species level, counted and pooled according to date, location, and species. Culex pipiens mosquitoes (including biotypes pipiens, molestus, and their hybrids), previously indicated as the primary WNV vector species in this region (9,13), were tested in pools of 50 mosquitoes. One mosquito pool was tested per sampling site for each date of collection.

### WNV detection

The preparation of samples for molecular detection was as follows: Tissue samples (brain, spleen, lung) of wild birds in amounts of 0.2 g or 50 individual mosquitoes (one pool) were placed in 2 mL microtubes and homogenized in 1 mL sterile phosphate-buffered saline for 5 min using a TissueLyser LT (Qiagen, Hilden, Germany) operating at 50 Hz. The homogenates were then centrifuged for 5 min at 2000·*g*, and the supernatant was used for RNA extraction. Tracheal swabs from wild birds were immersed in 0.75 mL of sterile phosphate-buffered saline, vortexed vigorously for 3 minutes, and then centrifuged for 5 min at 2000·g, and the supernatant was used for RNA extraction.

Mosquito pools and bird samples (tissues and tracheal swabs) were tested for WNV RNA presence by TaqMan-based one-step reverse transcription real-time PCR (RT-qPCR) that amplified both lineage 1 and 2 strains. Briefly, viral RNA was extracted using the commercial *ISOLATE II RNA Mini Kit* (Bioline, The Netherlands) according to the manufacturer’s instruction. One-step RT-qPCR was conducted using the commercial kit *RNA UltraSense™ One-Step qRT-PCR System* (Life Technologies Corporation) with the primers (forward WNproC-F10: 5’-CCTGTGTGAGCTGACAAACTTAGT-3’ and reverse WNproC-R153: 5’-GCGTTTTAGCATATTGACAGCC-3’) and probe (WNproC-probe 5’-FAM-CCTGGTTTCTTAGACATCGAGATCT-TAMRA- 3’) that targeted the nucleocapsid protein C gene regions of WNV, as described by Linke et al. [27]. Each reaction contained 15 μl of reaction mix containing 1 X RNA UltraSense reaction mix, 20 μM of each primer, 10 μM of the WNproC probe, 1 X ROX reference dye and 1 μl of RNA UltraSense enzyme mix. To this reaction mix, 5 μl of nucleic acid extract sample was added to make a final reaction volume of 20 μl. The thermocycling conditions were 15 min at 50°C and 2 min at 95°C, followed by 50 cycles of 15 s at 95°C and 50 s at 60°C.

### Human cases

Data from human cases used for the evaluation of WNV horse, bird and mosquito surveillance represent the number of clinical and laboratory confirmed West Nile positive cases reported to the European Centre for Disease Prevention and Control (ECDC) in both 2014 and 2015 (available at https://ecdc.europa.eu/en/table-cases-2014 and https://ecdc.europa.eu/en/table-cases-2015).

## Results

### Results of WNV surveillance during 2014

**Fig 2:**
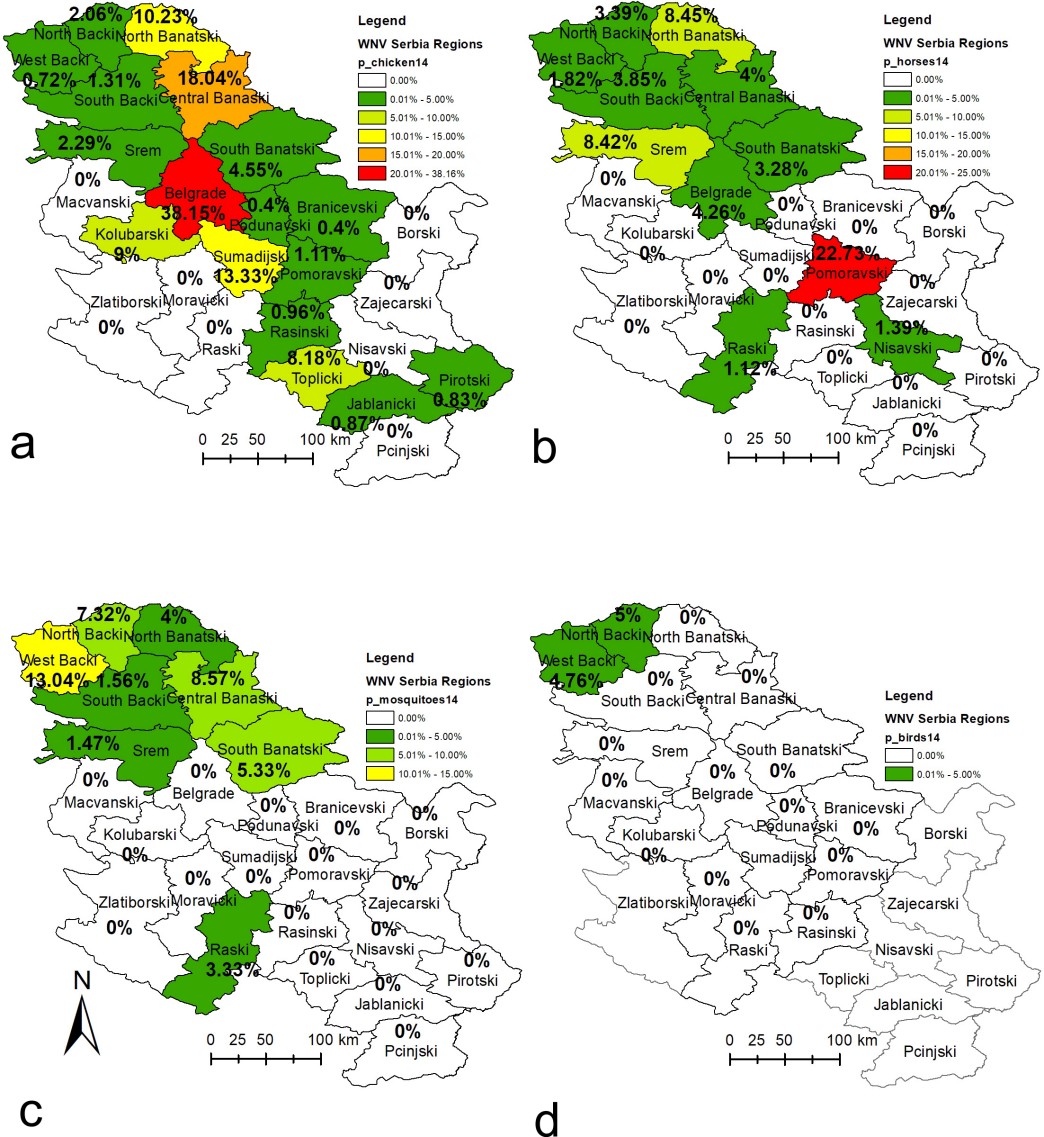
Distribution of WNV activity detected during the 2014 surveillance programme at the district level. Figs 2a-d show WNV infection rates as percentages (number positive samples/number of samples tested) for a) sentinel chickens b) sentinel horses, c) mosquito pools and d) wild birds. The basic administrative maps were extracted from the GADM database of Global Administrative Areas (www.gadm.org), version 2.8, November 2015, and later on changed according to the data presented in them.

**Fig 3:**
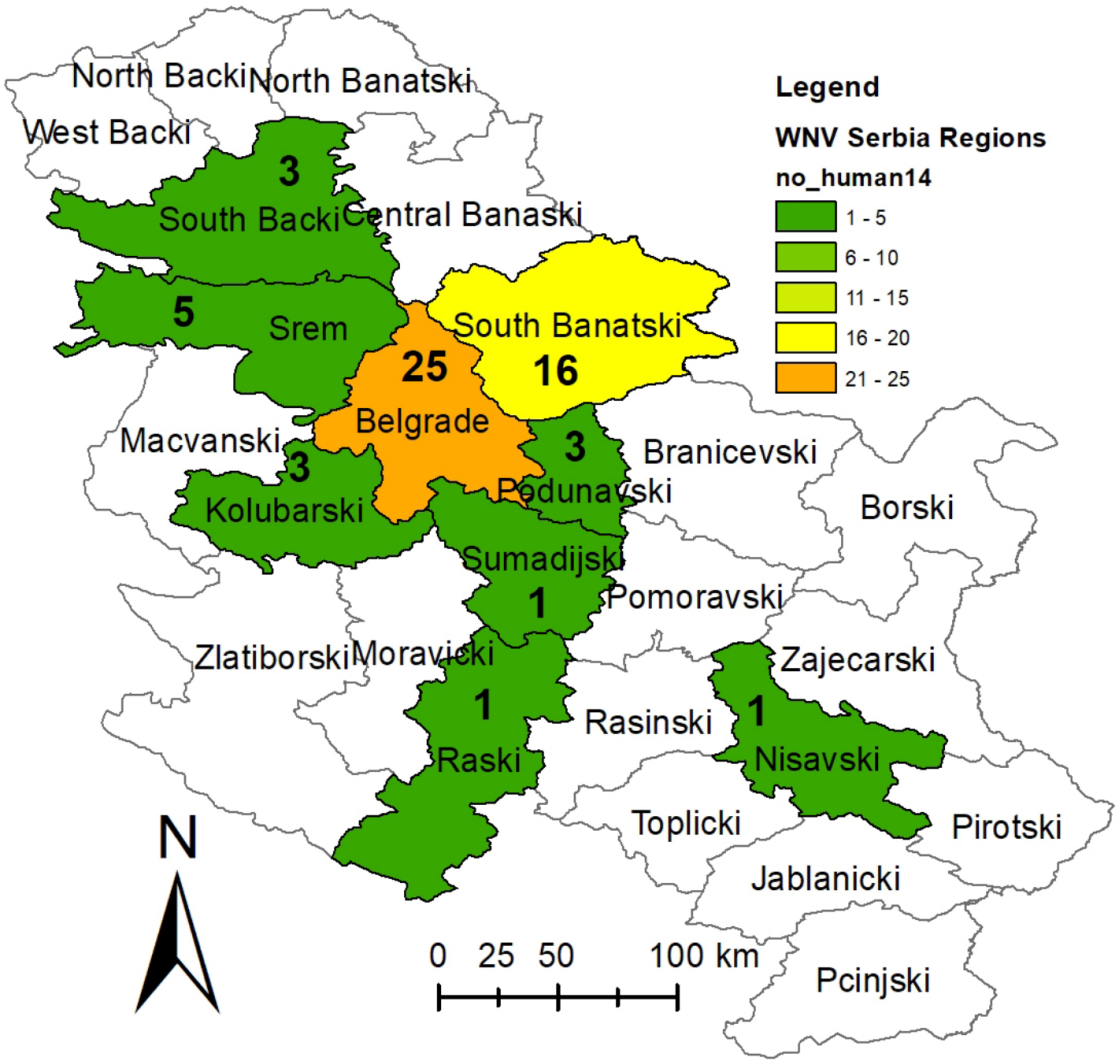
Distribution of human West Nile cases reported during the 2014 at the district level. Fig 3 shows the number of clinical and laboratory confirmed human West Nile cases reported to the ECDC (except for one clinical case in Raška and Nišava district that were not laboratory confirmed). The basic administrative maps were extracted from the GADM database of Global Administrative Areas (www.gadm.org), version 2.8, November 2015, and later on changed according to the data presented in them.

**Fig 4:**
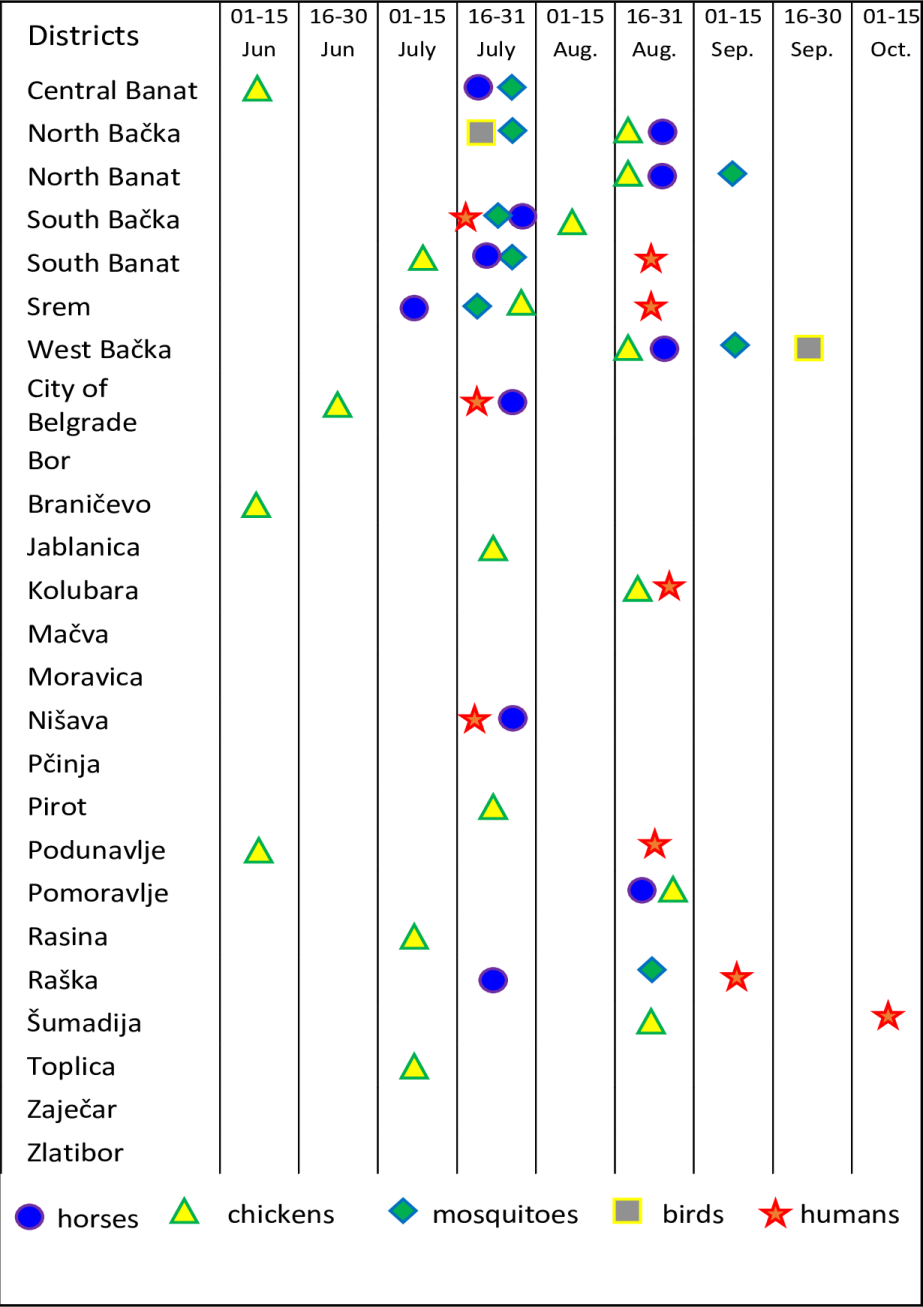
The temporal distribution of the first positive results detected during WNV surveillance and the time of the first human West Nile cases in 2014. The data for the first positive results obtained during WNV surveillance and the first reported human cases in 2014 are presented by symbols per district level. The top seven districts represent Vojvodina Province, followed by the other districts of Serbia sorted in alphabetical order.

A comparison of the surveillance data of all four tested cohorts (sentinel horses, backyard chickens, wild birds and vector mosquitoes) obtained during the WNV surveillance programme in Serbia in 2014 along with the human WNV-positive cases reported to the European Centre for Disease Prevention and Control (ECDC) in 2014 [28] are presented in Figs 2, 3 and 4. Detailed data for the results of the WNV surveillance programme in Serbia during 2014 and a comparison with the human WNV-positive cases reported to the ECDC in 2014 can be found in supporting information (S1 Table).

In the period June-August 2014 in Serbia, 2020 blood sera samples from 752 sentinel horses were tested, and seroconversion was detected in 2.57% (52/2020) of the tested samples and 6.91% (52/752) of the tested sentinel horses. Seroconversion was detected during all three months (June-August) in ascending order. As the programme progressed and with increasing vector activity, positive serological responses were determined in 0.53% (4/752), 1.38% (9/651) and 6.32% (39/617) of the tested sentinel horses during June, July and August, respectively. Seroconversion in the sentinel horses was detected in 11 (44%) out of 25 districts under surveillance. The number of districts in which the positive horses were detected was: one in June, five in July, and 11 in August. The percent of positively reactive / tested sentinel horses per district and the date of the first positive serological response per district are presented in Figs 2 and 4. Horses manifesting signs of neurological dysfunction were not observed during the surveillance programme.

In total, 3809 blood samples from backyard sentinel chickens were tested in the period June-September 2014, and seroconversion was detected in 5.75% (219/3809) of the samples. Seroconversion was detected during all four months of surveillance and showed an increasing trend during the programme period, with 4.37% (44/1006) in June, 3.02% (36/1191) in July, 8.91% (85/954) in August and 8.21% (54/658) in September. Positive serological responses were determined in backyard sentinel chickens in 17 of 25 districts (68%) under surveillance, with 7 districts in June, 6 districts in July, 11 districts in August, and in 8 districts in September. The percent of positively reactive / tested sentinel backyard chickens per district and the date of the first positive serological response per district are presented in Figs 2 and 4.

For direct surveillance of the virus, 995 pools of *Cx*. *pipiens* mosquitoes were tested, and WNV was confirmed in 2.31% (23/995) of the samples. The prevalence of WNV in mosquitoes was 3.1% (8/258) in July (4 positive on 16^th^ of July and 4 positive on the 30^th^ of July), 1.08% (3/278) in August (per one positive on 11^th^, 18^th^ and 30^th^ of August), and 9.68% (12/124) of the pool samples in September (5 positives on the 4^th^ of September and 7 positives on the 12^th^ of September). No WNV-positive mosquito pools were detected in May or June. The first WNV-positive mosquito pools were detected in the middle of July. West Nile virus-positive mosquitoes were detected in 8 (32%) out of 25 districts, with 5 districts in July, 3 districts in August, and in 5 districts in September. All districts, except the Raška District, where WNV-positive mosquito pools were detected are located in the Vojvodina Province. The percent of WNV-positive / tested mosquito pool samples per district and the date of the first WNV-positive mosquito pool sample detected per district are presented in Figs 2 and 4.

Among the 132 samples of dead and hunted wild birds, WNV was found in 1.52% (2/132) cases—in a dead cormorant (1/20; 5.0% of tested samples) on Palić Lake (North Bačka District) in July and in a hunted magpie (1/21; 4.76% tested sampes) in the West Bačka District in September 2014 (Fig 2D). Both detected WNV-positive wild birds were in the Vojvodina Province. WNV was not detected in any of the 736 tested pharyngeal swab samples of live wild birds. Wild bird samples were collected and tested from 16 out of the 25 districts in Serbia, and the percent of WNV-positive / tested wild bird samples per district and the date of the first WNV-positive wild bird sample detected per district are presented in Figs 2 and 4.

### Results of WNV surveillance during 2015

A high percentage of already seropositive horses, due to the WNV circulation in the previous years, as well as, errors occurred in determining the age of the backyard chickens during implementation of the WNV surveillance programme in 2014, influenced on the strategy used for the WNV surveillance programme in 2015. The basic changes in the WNV surveillance programme in 2015 included the exclusion of chickens as sentinel animals for serological surveillance and the testing of horses for the presence of IgM instead IgG antibodies.

**Fig 5:**
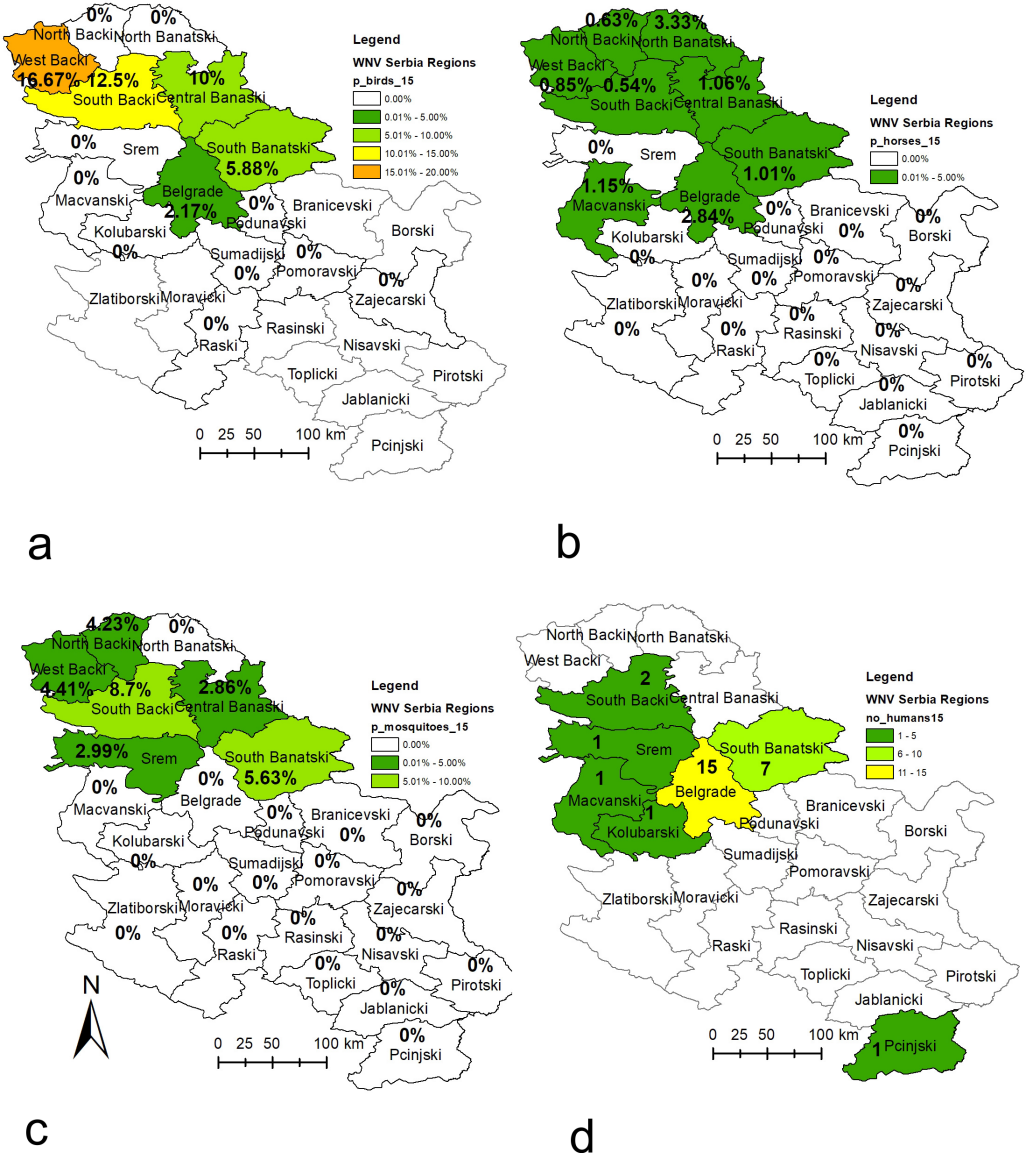
Distribution of WNV activity detected during the 2015 surveillance programme at the district level. Fig 5a-c show WNV infection rates as percentages (number positive samples/number of samples tested) for a) wild birds b) horses, and c) mosquito pools. Fig 5D presents the number of clinical and laboratory confirmed human West Nile cases reported to the ECDC. The basic administrative maps were extracted from the GADM database of Global Administrative Areas (www.gadm.org), version 2.8, November 2015, and later on changed according to the data presented in them.

**Fig 6:**
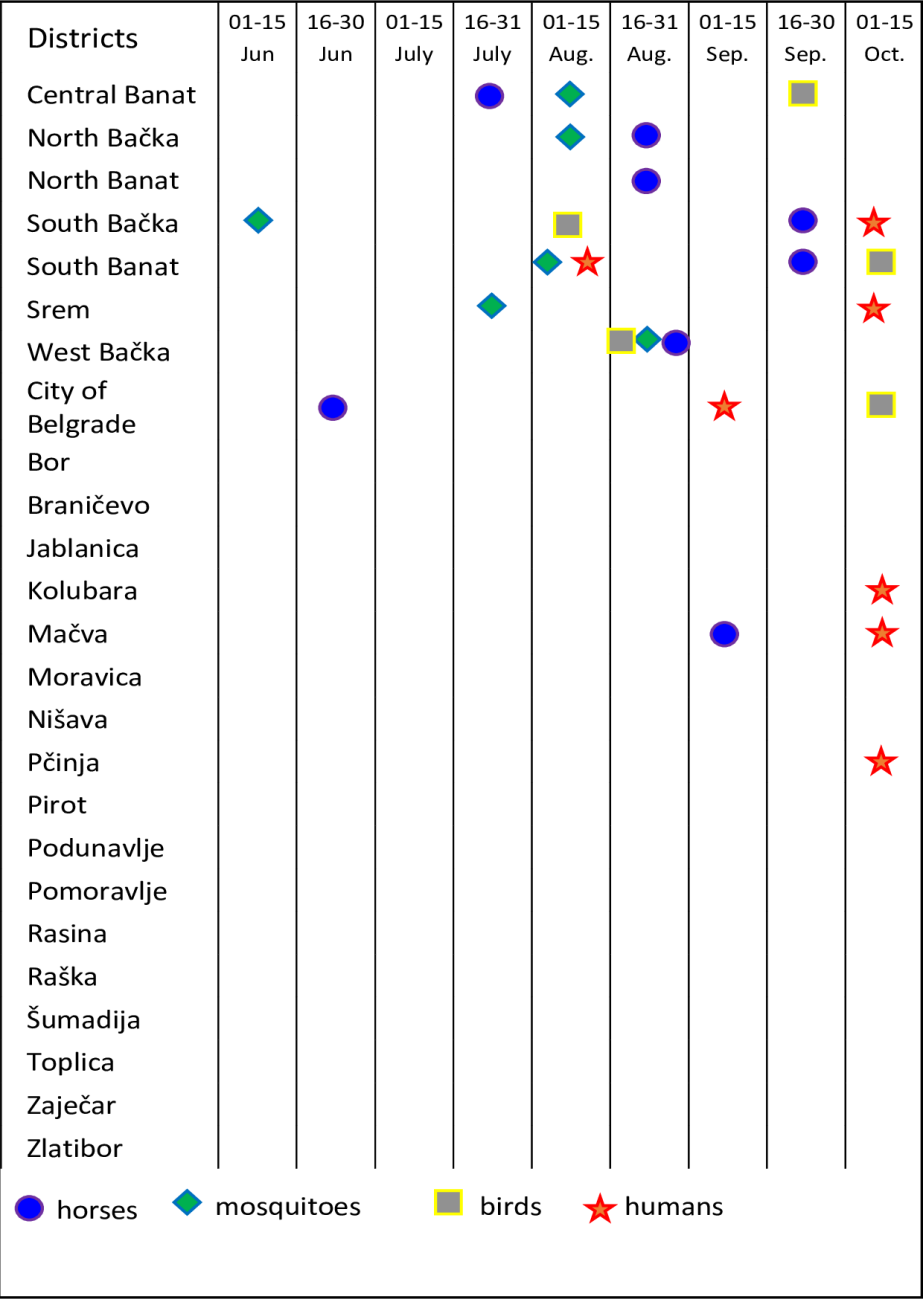
The temporal distribution of the first positive results detected during WNV surveillance and the time of the first West Nile human cases in 2015. The first positive results obtained during WNV surveillance and the first reported human West Nile cases in 2015 are represented by symbols per district level. The top seven districts represent the Vojvodina Province, followed by the other districts of Serbia sorted in alphabetical order.

A comparison of the surveillance data of all three tested cohorts (horses, wild birds and vector mosquitoes) obtained during the WNV surveillance programme in Serbia during 2015 with the human WNV-positive cases reported to the ECDC in 2015 [29] is presented in Figs 5 and 6. Detailed results of WNV surveillance programme in Serbia during 2015 and a comparison with the human WNV-positive cases reported to the ECDC in 2015 can be found in supporting information (S2 Table).

Between June and September 2015 in Serbia, positive serological responses (WNV IgM antibodies in blood sera) were detected in 0.53% (17/3238) of the tested horses. Positive serological responses were detected in all four tested months (June-September), with the highest prevalence in the period of most intense vector activity. The percentage of positively reacting animals in June was 0.13% (1/794), in July 0.12% (1/849), in August 1.11% (9/814), and in September 0.77% (6/781). Out of the 25 districts in Serbia, the number of districts in which the positive horses were detected was: one in June, one in July, five in August and four in September 2015. In total, positive serological responses in horses were detected in 8 (32%) out of the 25 districts in Serbia. The first positive serology result was detected in one horse from the Belgrade area in June 2015 (Fig 6). The percent of positively reacted / tested horses per district and the date of the first positive serological response detected per district are presented in Figs 5 and 6. Horses manifesting the signs of neurological dysfunction were not reported during the surveillance programme in 2015.

During the surveillance of viral presence in the environment, 956 pools of *Cx*. *pipiens* mosquitoes were tested from June to September 2015, and WNV was confirmed in 20 (2.09%) samples. The prevalence of WNV in mosquitoes increased after the first positive findings in June (0.38%; 1/264); 1.13% (3/266) mosquito samples tested positive in July, 4.92% (13/264) in August and 1.85% (3/162) in September. Positive mosquito samples were detected in 6 out of the 25 districts, with 8.7% (6/69) positive samples detected in South Bačka, 5.63% (4/71) in South Banat, 4.41% (3/68) in Western Bačka, 4.22% (3/71) in North Bačka, 2.99% (2/67) in Srem and 2.86% (2/70) in Central Banat (Fig 5). All districts where WNV-positive mosquito pools were detected are in the Vojvodina Province. The percent of WNV-positive / tested mosquito pool samples per district and the date of the first WNV-positive mosquito pool sample detected per district are presented in Figs 5 and 6.

Among the 183 samples of found dead wild birds, WNV was detected in 2 (1.09%) cases—in a hooded crow found in the city of Novi Sad in the South Bačka District in August and in a carrion crow found in the town of Pančevo in the South Banat District in September 2015. Among the 13 samples of hunted Eurasian magpies, WNV was detected in 3 (23.08%) cases, all of which were from the West Bačka District in September 2015. WNV was also detected in 3 (0.52%) out of the 524 tested pharyngeal swab samples of live-trapped wild birds from June-November 2015 (a hen harrier in the Central Banat District in September and 2 birds near Belgrade in October 2015). All detected WNV-positive wild birds were in 4 out of the 7 districts in the Vojvodina Province and around the City of Belgrade. The percent of WNV-positive / tested wild bird samples detected per district are presented in Figs 5 and 6.

## Discussion

We present the design and results of the WNV national surveillance programmes funded by the Veterinary Directorate of the Ministry of Agriculture and Environmental Protection conducted in 2014 and 2015 in Serbia. The main objectives for the programmes were the early detection of WNV circulating in the country, followed by the timely alerting of public health services and local authorities to increase both clinical and mosquito control preparedness. For those purposes, we developed the programmes considering different strategies previously used for WNV surveillance [9,21–25].

Sentinel chicken–based WNV surveillance systems have provided evidence of WNV transmission several weeks in advance of human cases in the USA [21,30]. Captive chickens, as sentinel animals, provide the most convenient source of blood for the monitoring of WNV transmission, being preferred blood-feeding hosts of *Cx*. *pipiens*, the primary WNV vector in this region [13,31,32]. However, we decided to include backyard chickens (instead of farmed and captive ones) as sentinels in 2014 based on the results of our study in 2013 (unpublished) and because it is less economically demanding than the establishment of a network of captive sentinels. In the unpublished study, the young chickens (hatched during 2013 to avoid seropositivity from previous years) from 5 chicken farms and ten backyards from the same area were tested for the presence of WNV IgG antibodies at the end of the vector activity season (November 2013). None of the farm chickens were seropositive, whereas 40–60% of the backyard chickens reacted positively. We suspect differences in levels of implemented biosecurity measures in a poultry farms and vector control intensity to be the main drivers of our findings. Difficulties in ageing the backyard chickens during surveillance efforts in 2014 made us exclude this method in 2015. However, the suitability of backyard chickens for early detection of WNV circulation was proven in Greece [33].

Although they are highly susceptible, horses cannot be used as sentinel animals in areas where vaccination of the horse population against WNV is widely used. In Serbia, horses are not vaccinated against WNV, and therefore they were included as sentinel animals in WNV surveillance. The same was true for the beginning of WNV surveillance in Emilia-Romagna, Italy, where animal surveillance consisted of passive and active surveillance of horses and active surveillance of wild birds and mosquitoes [34]. Based on the results of earlier studies on WNV seroprevalence in horses in Serbia [10–12], the initial testing for the presence of WNV IgG antibodies before the programme started in early spring 2014 encompassed a much larger horse population than was required for the surveillance, so that only seronegative horses would be included in the surveillance programme.

In addition to sentinel animals, in the Serbian surveillance programmes, direct monitoring of WNV presence was done by testing wild birds as WNV natural hosts and mosquitoes as virus vectors. The wild birds that were examined during the surveillance include species that are highly susceptible to WNV infection in various environmental conditions throughout the world as well as in the Serbian region [4,14,25,35] and have been efficiently used in WNV surveillance systems [25,36,37]. For the monitoring of WNV presence in vectors, only the mosquito species *Cx*. *pipiens* was tested because the results of previous studies suggested that this species is the most competent WNV vector in Serbia and the surrounding regions [13,31,32]. The WNV surveillance programmes based on these four surveillance pillars (horses, backyard chickens, wild birds and mosquitoes) were conducted during the period of most intensive vector activity during the year (May–September) [13].

The surveillance programme confirmed moderately intense circulation of WNV in 2014 in Serbia. The first WNV-positive mosquito pools were detected in the middle of July (July 16^th^), i.e., after the first positive serological responses in sentinel chickens (June 11^th^) and horses (July 15^th^). All districts, except the Raška District, where WNV-positive mosquito pools were detected are in the Vojvodina Province. Such a result could be attributed to the fact that the Vojvodina Province has previously been confirmed as a region with intensive WNV circulation in past years [10,12–14]. During wild bird surveillance in 2014, WNV presence was established in only 2 birds (in the Vojvodina Province). This, together with the fact that 736 pharyngeal swab samples from live wild birds tested negative for WNV, suggested low sensitivity for this part of the WNV surveillance programme in 2014.

Human cases were detected in Serbia in 2014, as expected, based on previous years. The characteristics of the 2014 outbreak were similar to those of the outbreak in 2012. In total, 76 clinical cases were reported, and 9 cases had fatal outcomes. Almost all cases were detected in the central and northern parts of the country, and 65 out of 76 (86%) of them were detected in just four districts (Belgrade, South Banat, South Bačka and Srem) [28]. The presence of WNV in the environment was first detected as positive serological responses in sentinel backyard chickens in the middle of June 2014 in 4 (16%) districts, including the Podunavlje District and the area of the City of Belgrade, where the majority of human cases were detected (35/76). The first positive serological response in horses in 2014 was detected on July 16 in the Srem District, a few days before the first human case in Serbia, but 5 weeks before the first human case in that particular district (this support our choice of districts as a units for good spatial resolution. The first WNV-positive mosquito samples (in 3 districts) and wild bird samples (in one district) were detected in the middle and at the end of July 2014. All except two human cases (74/76) were preceded by reports of WNV activity in their district of residence. Positive findings of WNV presence as detected by the implemented national surveillance system preceded human cases by three or more weeks in 6 out of the 9 districts in which the human cases were reported. In addition, positive surveillance results among the monitored domestic animals and / or mosquitoes were obtained in 10 more districts where human cases were not reported during 2014. Under these conditions, the surveillance showed good sensitivity for the detection of viral circulation, even at the enzootic level in the absence of human cases.

Although the WNV surveillance programme in 2014 proved very successful, it also highlighted two major drawbacks. The previous few years of viral circulation in the area resulted in a high percentage of already seropositive horses, so adequacy of the number of seronegative (sentinel) horses used in the programme in 2015 was questionable. Additionally, many errors occurred in determining the age of backyard chickens during implementation of the programme. Therefore, basic improvements of the WNV surveillance programme in 2015 were exclusion of chickens as sentinel animals for serological surveillance and the testing of horses for the presence of IgM instead IgG antibodies, as IgM antibodies indicate recent contact with the virus.

The results of the refined WNV surveillance programme in 2015 revealed a lower intensity of WNV circulation compared to 2014. Again, as in 2014, the regions with the most intensive WNV circulation in Serbia and where the majority of positive samples were detected were the Vojvodina Province and the Belgrade City area. Positive serological responses were detected in only 0.53% of the tested horses (2.57% in 2014) and in fewer districts (32%) than in 2014 (44%). The difference was not so large between the results of the entomological surveillance, where WNV was detected in 2.09% of the tested mosquito pools in 2015 compared to 2.31% in 2014. Positive mosquitoes were detected only in the Vojvodina Province. The first indication of WNV circulation in 2015 was observed in a mosquitoes (13 June), two months earlier than the first human case in Serbia and 3.5 months before the human case in the district of WNV positive mosquito first finding. Despite relatively high positivity of wild birds, viral detection was shifted towards the end of the season, and positive bird samples were obtained from only five districts. Similar to the mosquito surveillance data, all except one (from the City of Belgrade) detected WNV-positive wild birds were located in the Vojvodina Province.

The lower level of WNV circulation in the environment in 2015, was manifested in a much lower number of human cases. Almost all cases (27/28) were detected in the central and northern parts of the country, and 25 out of 28 (86%) of them were in four districts (the City of Belgrade, as well as the South Banat, South Bačka and Srem Districts of the Vojvodina Province) [29]. The declining trend of human cases and detections in horses could be partly explained by the fact that an intensive WNV circulation and WNV epidemic in both humans and animals have been present in Serbia for at least 6 years, and consequently, many people and animals had already been in contact with the virus, i.e., had an unapparent infection and had been immunized. Accordingly, the human population as well as susceptible animals were no longer totally naïve as was the case a few years ago.

Most of the human cases were preceded by the detection of WNV in animals and/or mosquitoes. Twenty six out of 28 human cases were preceded by surveillance reports of WNV activity in their district of residence. West Nile virus−positive animals and mosquitoes preceded human cases by two or more weeks in 4 out of the 7 districts in which the human cases were reported. Additionally, in one district, WNV presence in mosquito pool was detected one week earlier than the first human WNV case. In the two districts where one human WNV case was reported per district, there were no positive samples detected during surveillance. It might be possible that these two persons were infected in some other district of Serbia, but we have no data about their possible movement. The other possibility–that the surveillance programme was not sensitive enough to detect the lower intensity of WNV circulation–is less probable, considering that positive indications of WNV circulation were obtained in 4 more districts where no human cases were reported.

The districts of Vojvodina Province and the City of Belgrade have previously been identified as areas with high WNV circulation [10,12–14]. The possible explanation for that situation can be associated with geographical and climate features: the entire territory of Vojvodina Province and Belgrade City are located in the flatland area of up to 250 m altitude, characterized by a hot summer continental climate (*Dfa* climates), with an average temperature in the warmest month of ≥22°C as well as many slow water flows (Danube, Tisza and Sava rivers), small lakes and swamp areas that are favourite sites for shorter or longer stays for many migratory birds on their migration routes. It is highly probable that WNV was first introduced during the past few years by migratory birds into the aforementioned areas, which have abundant populations of *Cx*. *pipiens*, the most competent vector [13], which consequently became infected and further transmitted the virus to the resident birds as natural hosts [14]. It is likely that the infection will continue to present a significant problem and challenge for animal and human health.

The satisfactory results obtained in 2014 and 2015 are likely the consequence of the WNV surveillance programmes being designed and implemented with close collaboration among the veterinary service, ornithologists and entomologists. The results were continuously communicated by the Veterinary Directorate to the National Institute of Public Health. Then, the National Institute disseminated the surveillance results and information on citizen personal protection measures to the regional institutes of public health and district PH authorities. Vector and nuisance mosquito control is under the jurisdiction of the municipalities, and there is no national strategy for responding to early warnings about the risk of vector-borne disease outbreaks. Consequently, a database of mosquito treatments conducted in response to communicated alerts about WNV circulation does not exist, and we are not able to comment on the impact of control measures on WNV circulation. It seems that WNV cases in mosquitoes, birds, horses and humans and consequent outbreaks are clustered over a much larger area [38] than human cases and outbreaks associated with human-mosquito-human virus transmission (e.g., chikungunya, dengue). These types of transmission cycles pose a significant challenge in designing novel and adequate mosquito control measures [39]. In addition, the first detection of Usutu virus as a new zoonotic flavivirus in mosquitoes in Serbia [40] further complicate the situation and highlights the necessity for redesigning the existing WNV surveillance program and possibly the mosquito control strategy.

For the success of the programme, collaboration among veterinary and human health services as well as medical entomologists and the coordination and management of data at both national and local levels are essential in providing early warning and adequate protection of human and animal health. Based on the obtained results and anticipated continued intense circulation of WNV, further studies and continuous monitoring and surveillance of WNV infection and viral epidemiology in Serbia in the coming years will be of vital importance in order to further enhance early detection capacity and sensitivity and to improve the capacity to indicate the spatial distribution of the risk for WNV circulation.

## Supporting information

S1 Table Cumulative results of WNV surveillance program in Serbia during 2014, and comparison to the human WNV positive cases reported to the ECDC in 2014. (PDF)

S2 Table Cumulative results of WNV surveillance program in Serbia during 2015, and comparison to the human WNV positive cases reported to the ECDC in 2015. (PDF)

S3 Table Description of the sampling locations: Districts names, geographic coordinates and web links. (PDF)

S1 AJE Editorial Certificate (PDF)
