# Supplementary material for: Methodology and results of integrated WNV surveillance programmes in Serbia
Source: PLoS One. 2018 Apr 6;13(4):e0195439. doi: 10.1371/journal.pone.0195439 (PMC5889191; doi:10.1371/journal.pone.0195439)
Supplement: S1 Table — (PDF) [file pone.0195439.s001.pdf]

S1 Table: Cumulative results of WNV surveillance program in Serbia during 2014, and comparison to the human WNV positive cases reported to the ECDC in 2014

Districts (NUTS3)*	Sentinel horses-blood sera for anti-WNV IgG Ab			Sentinel chickens-blood sera for anti-WNV IgG Ab			Mosquitoes (Culex pipiens)			Wild birds (tissues and tracheal swabs)			Human cases***	
	Tested	Positive	First positive reported	Tested	Positive	First positive reported	Tested pools	Positive pools	First pos. pool reported	Tested	Positive	First positive reported	First case reported	Total No cases
Central Banat	100	4	17/07	255	46	15/06	70	6	16/07	23	0	-	-	-
North Bačka	118	4	30/08	194	4	28/08	82	6	16/07	20	1	29/07	-	-
North Banat	71	6	21/08	88	9	26/08	25	1	04/09	9	0	-	-	-
South Bačka	130	5	**21/07**	306	4	08/08	64	1	30/07	22	0	-	17/07	3 (5)
South Banat	122	4	**29/07**	220	10	**04/07**	75	4	**30/07**	6	0	-	29/08	16 (19)
Srem	95	8	**15/07**	261	6	**29/07**	68	1	**16/07**	13	0	-	22/08	5 (6)
West Bačka	110	2	23/08	140	1	23/08	23	3	04/09	21	1	21/09	-	-
City of Belgrade	47	2	NN**/07	270	103	**NN**/06**	59	0	-	88	0	-	17/07	25 (35)
Bor	66	0	-	120	0	-	15	0	-	-	-	-	-	-
Braničevo	60	0	-	250	1	15/06	70	0	-	65	0	-	-	-
Jablanica	88	0	-	115	1	24/07	25	0	-	-	-	-	-	-
Kolubara	12	0	-	100	9	**21/08**	27	0	-	71	0	-	22/08	3 (4)
Mačva	83	0	-	95	0	-	42	0	-	55	0	-	-	-
Moravica	86	0	-	112	0	-	25	0	-	127	0	-	-	-
Nišava	72	1	28/07	120	0	-	25	0	-	-	-	-	17/07	(1)
Pčinja	87	0	-	120	0	-	25	0	-	-	-	-	-	-
Pirot	90	0	-	120	1	24/07	25	0	-	-	-	-	-	-
Podunavlje	45	0	-	250	1	**11/06**	70	0	-	80	0	-	29/08	3 (4)
Pomoravlje	66	15	26/08	90	1	25/08	30	0	-	98	0	-	-	-
Rasina	81	0	-	104	1	05/07	30	0	-	100	0	-	-	-
Raška	87	1	**20/07**	89	0	-	30	1	**30/08**	70	0	-	10/09	(1)
Šumadija	39	0	-	90	12	**25/08**	20	0	-	-	-	-	02/10	1
Toplica	72	0	-	110	9	12/07	25	0	-	-	-	-	-	-
Zaječar	125	0	-	120	0	-	20	0	-	-	-	-	-	-
Zlatibor	68	0	-	70	0	-	25	0	-	-	-	-	-	-
**25**	**2020**	**52**		**3809**	**219**		**995**	**23**		**868**	**2**			**56 (76)**
%		***2.57***			***5.75***			***2.31***			***0.23***			

"-" not done and not detected

* The first seven (7) districts represents the Vojvodina Province (divided by double line from the other districts in Serbia – all presented by alphabetical order)

** The exact day of the first occurrence is not known just the month

*** Human cases reported to the ECDC (European Centre for Disease Prevention and Control) in 2014: laboratory confirmed cases (clinical cases) – available at: http://ecdc.europa.eu/en/healthtopics/west_nile_fever/West-Nile-fever-maps/Pages/2014-table.aspx

[yellow box] - positive results obtained during surveillance that preceded human infections at districts level

[red box] - positive results obtained during surveillance that didn't preceded human infections at districts level
