# Supplementary material for: Methodology and results of integrated WNV surveillance programmes in Serbia
Source: PLoS One. 2018 Apr 6;13(4):e0195439. doi: 10.1371/journal.pone.0195439 (PMC5889191; doi:10.1371/journal.pone.0195439)
Supplement: S2 Table — (PDF) [file pone.0195439.s002.pdf]

S2 Table: Cumulative results of WNV surveillance program in Serbia during 2015, and comparison to the human WNV positive cases reported to the ECDC in 2015

Districts (NUTS3)*	Horses-blood sera for anti-WNV IgM Ab			Mosquitoes (Culex pipiens)			Wild birds (tissues and tracheal swabs)			Human cases***	
	Tested	Positive	First positive reported	Tested pools	Positive pools	First pos. pool reported	Tested	Positive	First positive reported	First case reported	Total No cases
Central Banat	189	2	28/07	70	2	10/08	1	1	20/09	-	-
North Bačka	160	1	27/08	71	3	10/08	11	0	-	-	-
North Banat	120	4	25/08	22	0	-	5	0	-	-	-
South Bačka	186	1	**28/09**	69	6	**13/06**	8	1	**14/08**	05/10	2
South Banat	198	2	28/09	71	4	**04/08**	17	1	08/10	10/08	7
Srem	140	0	-	67	2	**21/07**	11	0	-	05/10	1
West Bačka	234	2	28/08	68	3	30/08	18	3	23/08	-	-
City of Belgrade	141	4	**NN**/06**	40	0	-	93	2	NN**/10	14/09	15
Bor	108	0	-	20	0	-	0	0	-	-	-
Braničevo	84	0	-	25	0	-	0	0	-	-	-
Jablanica	120	0	-	20	0	-	0	0	-	-	-
Kolubara	148	0	-	15	0	-	200	0	-	**02/11**	1
Mačva	87	1	**09/09**	9	0	-	53	0	-	02/11	1
Moravica	123	0	-	20	0	-	0	0	-	-	-
Nišava	120	0	-	20	0	-	0	0	-	-	-
Pčinja	120	0	-	19	0	-	0	0	-	**05/10**	1
Pirot	120	0	-	20	0	-	0	0	-	-	-
Podunavlje	97	0	-	70	0	-	82	0	-	-	-
Pomoravlje	118	0	-	20	0	-	3	0	-	-	-
Rasina	75	0	-	20	0	-	0	0	-	-	-
Raška	91	0	-	70	0	-	110	0	-	-	-
Šumadija	135	0	-	70	0	-	107	0	-	-	-
Toplica	120	0	-	20	0	-	0	0	-	-	-
Zaječar	102	0	-	20	0	-	1	0	-	-	-
Zlatibor	102	0	-	20	0	-	0	0	-	-	-
**25**	**3238**	**17**		**956**	**20**		**720**	**8**			**28**
%		***0.53***			***2.09***			***1.11***			

"-" not done and not detected

* The first seven (7) districts represents the Vojvodina Province (divided by double line from the other districts in Serbia – all presented by alphabetical order)

** The exact day of the first occurrence is not known just the month

*** Human cases reported to the ECDC in 2015: laboratory confirmed cases (clinical cases) – available at: https://ecdc.europa.eu/en/table-cases-2015

[yellow] - positive results obtained during surveillance that preceded human infections at districts level

[red] - human infections at the district level where no previous positive findings were obtained during surveillance
