# Supplementary material for: Methodology and results of integrated WNV surveillance programmes in Serbia
Source: PLoS One. 2018 Apr 6;13(4):e0195439. doi: 10.1371/journal.pone.0195439 (PMC5889191; doi:10.1371/journal.pone.0195439)
Supplement: S3 Table — (PDF) [file pone.0195439.s003.pdf]

S3 Table: Description of the sampling locations: Districts names, geographic coordinates and web links

Districts (NUTS3)*		Geographic coordinates (the center of the district)	Web links for basic decription of the Districts
Vojvodina Province	Central Banat	45.4788° N, 20.6083° E	https://en.wikipedia.org/wiki/Central_Banat_District
	North Bačka	45.9803° N, 19.5907° E	https://en.wikipedia.org/wiki/North_Ba%C4%8Dka_District
	North Banat	45.9068° N, 19.9993° E	https://en.wikipedia.org/wiki/North_Banat_District
	South Bačka	45.4890° N, 19.6976° E	https://en.wikipedia.org/wiki/South_Ba%C4%8Dka_District
	South Banat	45.0027° N, 21.0543° E	https://en.wikipedia.org/wiki/South_Banat_District
	Srem	45.0029° N, 19.8014° E	https://en.wikipedia.org/wiki/Srem_District
	West Bačka	45.7355° N, 19.1897° E	https://en.wikipedia.org/wiki/West_Ba%C4%8Dka_District
Central Serbia	City of Belgrade	44.5850° N, 20.3964° E	https://en.wikipedia.org/wiki/Belgrade
	Bor	44.3702° N, 22.2857° E	https://en.wikipedia.org/wiki/Bor_District
	Braničevo	44.5298° N, 21.4910° E	https://en.wikipedia.org/wiki/Brani%C4%8Devo_District
	Jablanica	42.9482° N, 21.8129° E	https://en.wikipedia.org/wiki/Jablanica_District
	Kolubara	44.3510° N, 20.0004° E	https://en.wikipedia.org/wiki/Kolubara_District
	Mačva	44.5925° N, 19.5082° E	https://en.wikipedia.org/wiki/Ma%C4%8Dva_District
	Moravica	43.8415° N, 20.2905° E	https://en.wikipedia.org/wiki/Moravica_District
	Nišava	43.3739° N, 21.9322° E	https://en.wikipedia.org/wiki/Ni%C5%A1ava_District
	Pčinja	42.5836° N, 22.1430° E	https://en.wikipedia.org/wiki/P%C4%8Dinja_District
	Pirot	43.0874° N, 22.5983° E	https://en.wikipedia.org/wiki/Pirot_District
	Podunavlje	44.4729° N, 20.9901° E	https://en.wikipedia.org/wiki/Podunavlje_District
	Pomoravlje	43.9591° N, 21.2714° E	https://en.wikipedia.org/wiki/Pomoravlje_District
	Rasina	43.5264° N, 21.1588° E	https://en.wikipedia.org/wiki/Rasina_District
	Raška	43.3373° N, 20.5734° E	https://en.wikipedia.org/wiki/Ra%C5%A1ka_District
	Šumadija	44.2051° N, 20.7857° E	https://en.wikipedia.org/wiki/%C5%A0umadija_District
	Toplica	43.1907° N, 21.3408° E	https://en.wikipedia.org/wiki/Toplica_District
	Zaječar	43.8039° N, 22.0538° E	https://en.wikipedia.org/wiki/Zaje%C4%8Dar_District
	Zlatibor	43.6454° N, 19.7101° E	https://en.wikipedia.org/wiki/Zlatibor_District
